# Numerical investigation of the effect of carotid bifurcation stenosis degree on pulsatility characteristics

**DOI:** 10.3389/fphys.2023.1169198

**Published:** 2023-07-06

**Authors:** Chao Liu, Gao Wu, Jianxin Xu, Qingtai Xiao, Hua Wang

**Affiliations:** ^1^ Affiliated Hospital of Kunming University of Science and Technology, Kunming, China; ^2^ First People’s Hospital of Yunnan Province, Kunming, China; ^3^ Faculty of Metallurgical and Energy Engineering, Kunming University of Science and Technology, Kunming, China

**Keywords:** carotid bifurcation, stenosis rate, wall shear stress, vortex intensity, biological mass and heat transfer

## Abstract

Arterial bifurcations are regions that are susceptible to hemodynamic effects and thrombus formation. In the current study, the hemodynamic effects of a simplified 3D model of an arterial bifurcation were simulated using the commercial computational fluid dynamics software FLUENT. The non-Newtonian properties of blood were modeled using the Carreau model, and the pulsation dynamics and heat transfer characteristics of blood at different degrees of stenosis in the arterial bifurcation were analyzed. The results indicate that arterial stenosis caused by a thrombus when the pulsation velocity reaches its peak has an essential impact on blood transport. The stenosis of the bifurcation increases the peak pulsatile flow pressure drop, and each 0.5 mm stenosis of the arterial bifurcation increases the mean wall shear stress of the bifurcated segment by approximately 0.25 Pa. From the heat transfer perspective, arterial stenosis has little effect on the heat transfer coefficient. The heat transfer coefficient measured inside the bifurcation is much larger than that measured outside the bifurcation. The stenosis of the arterial bifurcation causes an increase in the mean velocity of the arterial cross-section, and the volume-averaged absolute vorticity is introduced to quantify the secondary flow effect during the pulsation cycle, where the arterial stenosis causes an increase in the mean absolute vorticity at pulsation velocity and accelerates the decay of the vorticity at uniform velocity. In this paper, the hemodynamics of carotid bifurcation pulsation is analyzed in conjunction with flow field properties to reveal the flow field dynamics factors and heat transfer characteristics of local stenosis of the carotid bifurcation and to conduct an exploratory study for the diagnosis and treatment of carotid bifurcation thrombosis.

## 1 Introduction

Arterial disease is a risk factor that affects the entire vascular system. Atherosclerotic plaques usually occur in specific geometric regions, such as the internal curvature of the aortic arch and near arterial bifurcations, particularly in the carotid bifurcation region (Spanos et al., 2017). Carotid bifurcation geometry is considered a marker of atherosclerosis. With the development of computational fluid dynamics, the influence of various geometric factors on local hemodynamic changes was shown to be related to the development of atherosclerosis (Sitzer et al., 2003).

The influence of hemodynamic factors on the progression of the atherosclerotic disease has been widely noted (Caplan and Baker, 1980; Friedman et al., 1983). The variable and irregular shape of blood vessels leads to localized rotational movements of blood particles in some regions of the vessels, which is detrimental to blood transport, and blood cells are prone to cellular accumulation in regions of localized rotation, resulting in easy formation of thrombi in these areas (Aziz et al., 2017). A study by Ariane et al. (Ariane et al., 2018) reported that the elasticity of thrombi was related to the time elapsed after thrombosis. The longer the time elapsed after thrombosis, the less elastic the thrombus. The longer the time experienced after thrombus formation, the less elastic the thrombus is. After the wall shear of the vessel is less than 0.4 Pa, leukocytes tend to accumulate at the wall and thus form thrombi, as reported by Kien et al., 2008. In cases where the flow is more complex, such as vascular bifurcation, the local flow characteristics of the blood are significantly altered, and it is easier to form thrombi at the bifurcation (Lee et al., 2022). Changes in the blood flow state can cause sudden changes in the shear forces generated by the blood for the wall. The blood flow state becomes more complex, making it easy to create local turbulence, vortices, and secondary flow, which can adversely affect the intima of the blood vessels and cause damage to the endothelium (Jewgenow et al., 2019).

The formation process of thrombosis can be analyzed by combining several methods, and with the development of computer technology, computational fluid dynamics provides new research methods to study thrombosis. Many computational models have been developed, such as the one by Bharadvaj et al. (Bharadvaj et al., 1982a; Bharadvaj et al., 1982b). In computational fluid dynamics, the process of thrombus formation can be analyzed by combining several methods in which platelets approaching a damaged vessel wall surface activate and adhere to that wall surface, a phenomenon related to the physiological mechanisms of platelets. Kamada et al., 2010 suggested that the Reynolds number is related to the properties of blood and can be used to indicate the state of flow; blood flow in a vessel with different Reynolds numbers has different aspect ratios for thrombus formation in the damaged wall; the higher the Reynolds number, the upper part of the thrombus formation on the wall is more easily dispersed by the blood flow, thus elongating the lateral dimension of the thrombus. After thrombus formation, blood will create eddies upstream of the thrombus, further increasing the degree of platelet aggregation upstream, which may trigger thrombus growth and the construction of a new thrombus. After the thrombus moves to the low-velocity zone, the length of the thrombus determines the time for the thrombus to break away from the zone; the longer the thrombus, the easier it is to break away from the low-velocity zone, and the shorter the thrombus, the less likely it is to flow out of the low-velocity zone. Thrombosis can be treated by targeted delivery of thrombolytic agents through catheters or surgical thrombus removal (Zhang et al., 2017). When the delivery catheter is poorly positioned, it may result in a poor concentration of thrombolytic agent at the location where the thrombus exists, failure to achieve thrombolysis, and may be accompanied by bloodstream infection. After surgical implantation of a stent or bypass graft into a vessel, wall shear is high at the connection between the placement and the vessel wall. Increased upstream shear can lead to cytokine activation downstream, cell aggregation, and susceptibility to platelet adhesion (Rahman et al., 2018). When a blood vessel branches at a downstream location, a shunt of blood occurs at the bifurcation. Due to certain factors, a thrombus may form in the bifurcated vessel. The diameter of the vessel upstream of the bifurcation of the vital artery is approximately 5.5–9 mm, and at the bifurcation, the vessel diameter is approximately 5.6–9.8 mm (Thomas et al., 2005). In angiographic images, the common artery at the carotid bifurcation is approximately 7 mm in diameter, and the common and internal carotid arteries are susceptible to stroke due to thrombosis (Lee et al., 2008). The thrombus at the bifurcation of the carotid artery will produce abnormal shear forces on the wall. As the size and density of the thrombus change, the flow velocity at the bifurcation will also change. The thrombus may come into contact with the bifurcation of the vessel during the movement of the thrombus, and when the two come into contact due to the irregular shape of the bifurcation, resulting in a higher stress on the thrombus (Wells et al., 1996). Amiri et al. (Amiri et al., 2019) simulated femoral artery blood flow using fluid-solid interaction and investigated the effect of Newtonian and non-Newtonian blood flow on the elastic vessel wall. The results showed negligible displacement of the vessel wall due to blood flow pulsation and significant differences in wall shear stresses calculated by the Newtonian and non-Newtonian fluid models, indicating that the assumptions of the Newtonian fluid model do not yield the desired results.

Arterial thrombosis is a common disease, and the cause of the formation of certain thrombi may be related to various factors. Due to the influence of hemodynamics, it is necessary to analyze the possible locations of thrombus formation in a specific vessel and the causes of formation. The study of hemodynamics in blood vessels can give possible causes of formation in the direction of hydrodynamics for common clinical thrombi, which is of specific guidance for clinical analysis. In the current study, the pattern of motion of pulsatile flow in arterial bifurcation vessels was investigated using changes in vessel morphology in local stenosis assuming thrombus formation. Numerical simulations of arterial bifurcation flow were performed using the Ansys Fluent commercial fluid calculation software, which treats blood as a non-Newtonian fluid and models blood using the Carreau model. The pulsation condition passages were configured using simplified entrance boundary conditions to study the pulsatile blood flow in human bifurcation vessels analytically. To find the various factors affecting the local flow characteristics of the bifurcation by simulating the pulsatile flow of blood in an actual bifurcated vessel, the flow factors that produce atherosclerosis will be analytically investigated. In this study, the bifurcation vessels of the carotid artery will be used as the object of study. Numerical simulations of computational fluid dynamics (CFD) will be used to study and analyze the pulsatile flow characteristics of the bifurcation vessels concerning the physiological characteristics of blood and blood vessels and to examine in complete detail the factors that may affect the local aspects of the pulsatile bifurcation flow.

## 2 Simulation method

### 2.1 Computational geometry models

The relationship between carotid bifurcation geometry, flow characteristics and atherosclerosis has been investigated using carotid bifurcation models. The Y-Shaped Average Human Carotid Bifurcation (Y-AHCB) model was constructed by Bharadvaj (1982) (Bharadvaj et al., 1982a; Bharadvaj et al., 1982b) and others based on angiographic photographs of patients. The model was established based on a large amount of statistical analysis of human anatomical data. Therefore, it can more accurately reflect the actual physiological geometric characteristics of human carotid arteries. Therefore, this study’s simplified carotid artery model should be based on the Y-AHCB model.

**FIGURE 1:**
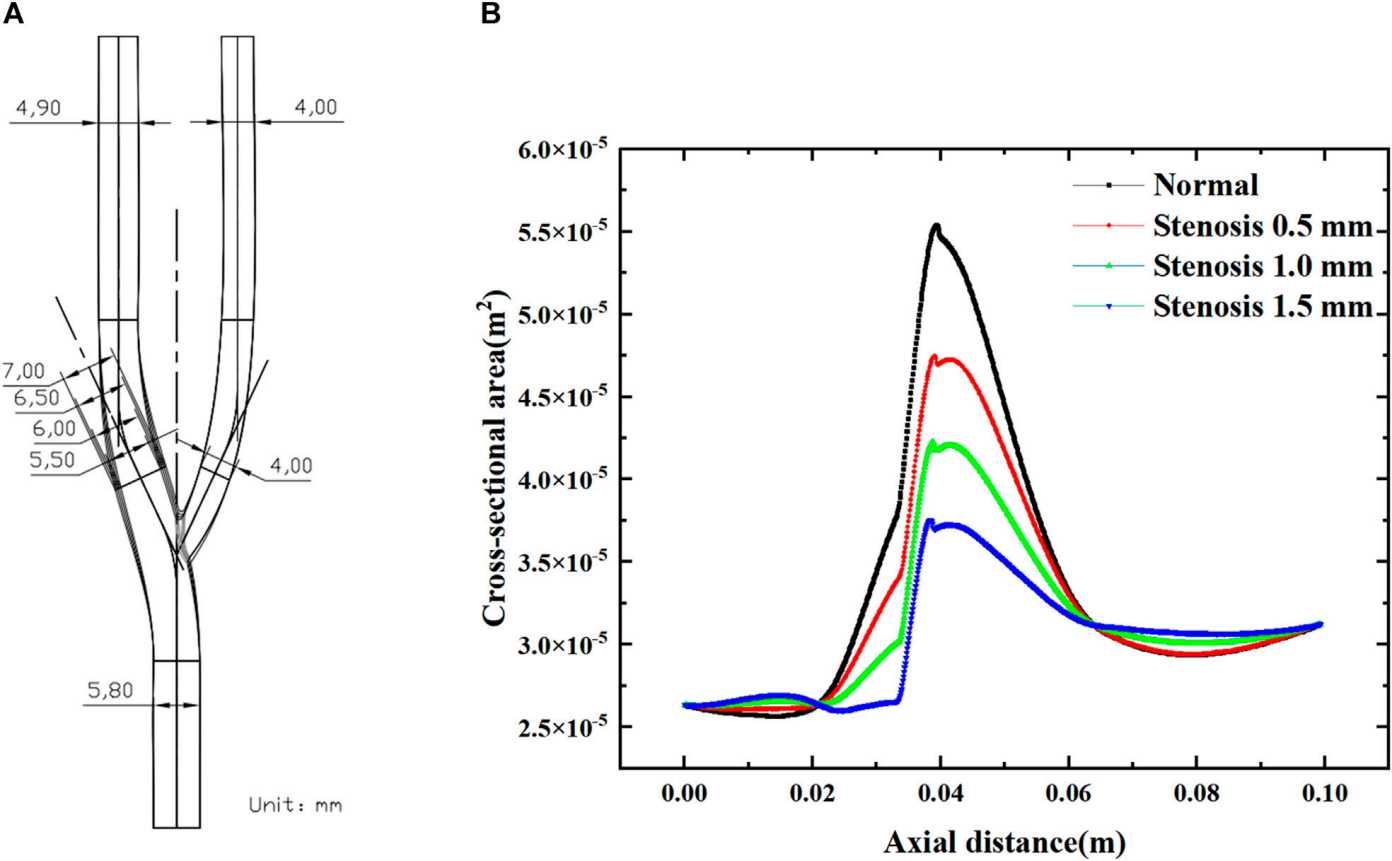
Numerical simulation geometry model. **(A)** Normal carotid artery and tenosis 0.5–1.5 mm carotid artery. **(B)** The cross-sectional area of bifurcated vessels along the axial direction. **(B)** shows the variation in cross-sectional area along the axial direction for the four arterial bifurcation models, with the degree of stenosis having a significant effect on the cross-sectional area of the bifurcated segment (0.02–0.065 m). The increase in the degree of stenosis leads to a decrease in the local cross-sectional area. For each 0.5 mm increase in stenosis, the maximum cross-sectional area at the bifurcation decreased by approximately 7.5 × 10^-4^ m^2^.

This study established a three-dimensional simplified geometric model of the carotid artery and three bifurcation stenosis models with stenoses of 0.5 mm–1.5 mm, as shown in Figure 1. In Figure 1A, the bifurcation angle was selected as 50°. In Figures 1A, a schematic diagram of the carotid bifurcation geometry model is shown, using a total of four geometric models, each narrowing the bifurcation by 0.5 mm compared to the normal vessel bifurcation structure.

### 2.2 Blood physical properties

For CFD calculations, blood is assumed to be an isotropic, incompressible fluid with a constant density *ρ* = 1,060 kg/m^3^ and a temperature T = 310 K. The physical properties of blood are shown in Table 1, based on the physiological properties of blood. Blood vessels are thin-walled, rigid, non-permeable, non-slip inelastic solids.

**TABLE 1:** Blood physical properties.

Property	Blood property at 310 K
Density	1060 kg/m^3^
Specific heat	3770J/kg.K
Thermal conductivity	0.51W/m.K

Blood is a special kind of fluid, a non-Newtonian fluid composed of plasma, red blood cells, white blood cells, platelets, *etc.* Under quasi-static conditions, the stress-strain relationship of blood is nonlinear, and there is yield stress. The lower the shear rate, the stronger the nonlinearity. Under dynamic conditions, blood is a viscoelastic fluid. Some empirical or semi-empirical intrinsic structure relations have been established for this. However, none of them can qualitatively satisfy the available experimental results. It has been proved by numerous experiments that blood can be regarded as a Newtonian fluid when the shear rate is not too low, but there is a significant error when the shear rate is negligible. In contrast, the Carreau model (Jahangiri et al., 2015; Jahangiri et al., 2017) is consistent with the experiments over a wide range of shear rates. The model in Table 2 was used to simulate the non-Newtonian behavior of the fluid.

**TABLE 2:** Carreau’s non-Newtonian model.

Non-Newtonian model	Expression
Carreau	$\begin{array}{c} \begin{array}{c} \mu =\mu_{\infty }+\left(\mu_{0}-\mu_{\infty }\right){\left(1+A{\left|\dot{\gamma }\right|}^{2}\right)}^{n}\\ A=10.976,n=-0.3216,\mu_{\infty }=0.0033,\mu_{0}=0.056 \end{array} \end{array}$ (1)

### 2.3 Mesh independence test

**FIGURE 2:**
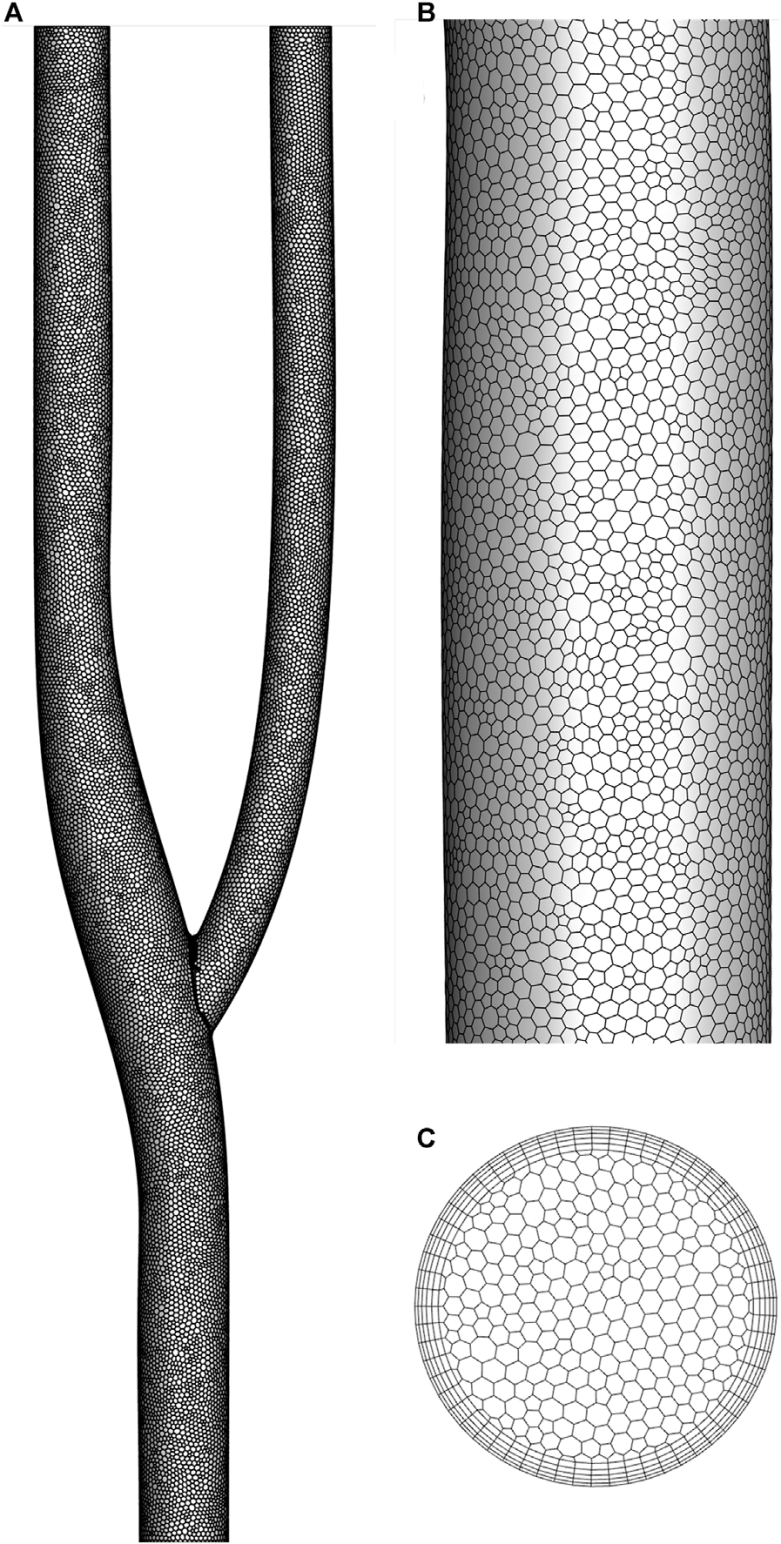
Detail of mesh. **(A)** Mesh generation. **(B)** Mesh detail. **(C)** Cross-section mesh.

Figure 2 shows the discrete method of the arterial bifurcation geometry model. A polyhedral mesh discretizes the entire computational domain for complex flow fields. The number of meshes is less than other types of meshes under the condition that the computational accuracy is guaranteed. The cross-sectional mesh shows the meshing of the boundary layer, encrypted using 5-layer mesh. The near-wall grid is encrypted to ensure the dimensionless distance y+<1 for capturing fluid interactions in the viscous sublayer.

$$\begin{array}{c} y^{+}=\frac{yu_{\tau }}{\nu } \end{array}$$ (2)

where y is the distance from the wall, u_τ is the friction velocity, and ν is the kinematic viscosity.

The mesh independence was verified using the pressure drop in the arterial bifurcation fluid region at a flow velocity of 0.5 m/s. Four groups of grids from Table 3 were used for the grid independence test, respectively. As shown in Table 3, the pressure drop increases with the grid number. The change of pressure drop is about 1 Pa when the grid number reaches 1114679 and continues to increase the grid number, indicating that the increase of grid number has little effect on the calculation results. The following calculation is performed using 1114679 cells.

**TABLE 3:** Mesh independence verification.

Number of cells	Pressure drop (Pa)
286988	35
588365	39
1114679	44
1634182	45

### 2.4 Governing equations

In this study, blood is assumed to be an incompressible, non-Newtonian fluid in a zero-gravity laminar flow state. The Navier-Stokes control equations, including the conservation of mass and momentum, are as follows (Barrett et al., 2015).

Continuity equation

$$\begin{array}{c} \frac{\partial \left(\rho u_{i}\right)}{\partial x_{i}}=0 \end{array}$$ (3)

Momentum equation

$$\begin{array}{c} \frac{\partial \left(\rho u_{i}\right)}{\partial t}+\frac{\partial \left(\rho u_{i}u_{j}\right)}{\partial x_{j}}+\frac{\partial p}{\partial x_{i}}=\frac{\partial }{\partial x_{i}}\left(\mu \frac{\partial \left(u_{i}\right)}{\partial x_{j}}-\rho \bar{u_{i}^{\prime }u_{j}^{\prime }}\right) \end{array}$$ (4)

Energy equation

$$\begin{array}{c} \frac{\partial \left(\rho T\right)}{\partial t}+\frac{\partial \left(\rho u_{i}T\right)}{\partial x_{i}}=\frac{\partial }{\partial x_{i}}\left(\frac{\lambda }{c_{p}}\frac{\partial T}{\partial x_{i}}\right) \end{array}$$ (5)

### 2.5 Boundary Conditions.

**FIGURE 3:**
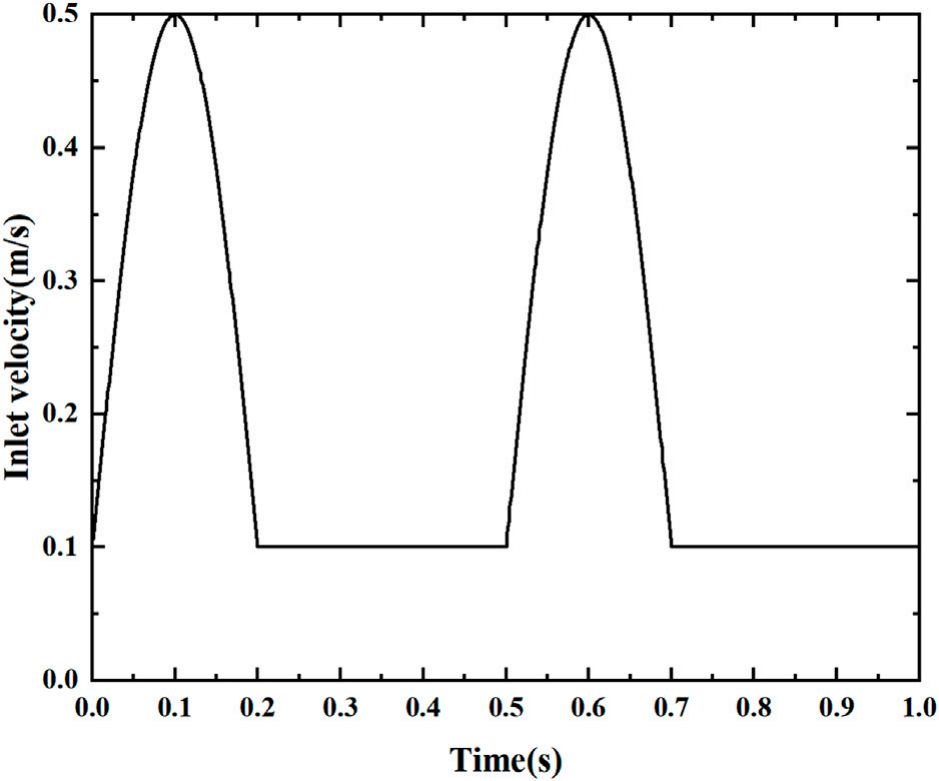
Used velocity pulse.

The fluid temperature at the arterial inlet is 310°C, with a vessel density *ρ* = 1,060 kg/m^3^, specific heat Cp = 3700 J/kg.K, thermal conductivity *λ* = 0.51 W/m.K, and heat flow at the wall of −1000 W/m^2^, using no-slip boundary conditions. Based on Sinnott’s study (Sinnott et al., 2006), a simplified pulsation velocity at the entrance was used, as shown in Figure 3. The periodic velocity inlet condition was defined using a user-defined function (UDF), as shown in Eq. 6. Since the blood flow velocity shows a significant periodic pulsation characteristic. The total computation time is 1s, characterizing the effect of two cycles to study the pulsation characteristics, dividing the entire simulation into 500 time steps, each with an interval ∆t = 0.0002 s. To obtain convergent periodic computational results, each stenosis model is computed for more than two consecutive cycles until the flow field reaches the periodic characteristics. The numerical simulation of the pulsatile blood flow field was completed using Fluent, a commercial computational fluid dynamics software. Pressure-velocity coupling was achieved using the SIMPLE algorithm. The gradients were calculated by a discretization method based on least squares units. The spatial discretization of pressure, momentum and energy was obtained by a second-order upwind scheme. The residual criterion for the energy equation is less than 10^–8^, while those for the other equations are set to less than 10^–6^.

$$\begin{array}{c} V_{inlet}\left(t\right)=\left\{\begin{array}{c} 0.1+0.4\times \sin\left(15.7\times t\right):0.5n<t\leq 0.5n+0.2\\ 0.1:0.5n+0.2<t\leq 0.5\left(n+1\right) \end{array}\right. \end{array}$$ (6)

### 2.6 Data reduction

Wall shear stress $\tau_{\omega }$ is calculated by Eq. 7:

$$\begin{array}{c} \tau_{\omega }=\mu \frac{\partial u}{\partial n} \end{array}$$ (7)

μ is the dynamic viscosity, u indicates the flow velocity, and n is the x, y, and z directions.

The axially averaged wall shear stress $\bar{\tau_{\omega y}}$ is calculated by Eq. 8:

$$\begin{array}{c} \bar{{\tau_{\omega }}_{y}}=\frac{1}{A\left(y\right)}\int \tau_{\omega }dA\left(y\right) \end{array}$$ (8)

A(y) is the area of the wall in the axial position.

The average wall shear stress $\bar{\tau_{\omega }}$ in the bifurcated section is calculated by Eq. 9:

$$\begin{array}{c} \bar{\tau_{\omega }}=\frac{1}{A}\int \tau_{\omega }dA \end{array}$$ (9)

A is the wall area of the bifurcated segment (axial position 0.02–0.065 m interval).

The surface local heat transfer coefficient h of the arterial bifurcation is defined as:

$$\begin{array}{c} h=\frac{q}{T_{w}-T_{f}} \end{array}$$ (10)

q is the heat flux at the wall, T_w_ is the wall temperature, and T_f_ is the mainstream fluid temperature.

The axial average heat transfer coefficient $\bar{h}$ is calculated by Eq. 11:

$$\begin{array}{c} \bar{h}=\frac{1}{A\left(y\right)}\int hdA\left(y\right) \end{array}$$ (11)

The longitudinal absolute vortex flux is defined as:

$$\begin{array}{c} \left|\omega_{y}\right|=\left|\frac{\partial w}{\partial x}-\frac{\partial u}{\partial z}\right| \end{array}$$ (12)

The volume-averaged absolute vortex flux is:

$$\begin{array}{c} {\left|\omega_{y}\right|}_{V}=\frac{1}{V}\int_{V}\left.{\left|\omega \right.}_{y}\right|dV \end{array}$$ (13)

V is the volume of the entire computational domain.

## 3 Results and discussion

### 3.1 Pressure drop variation with pulsation cycle

**FIGURE 4:**
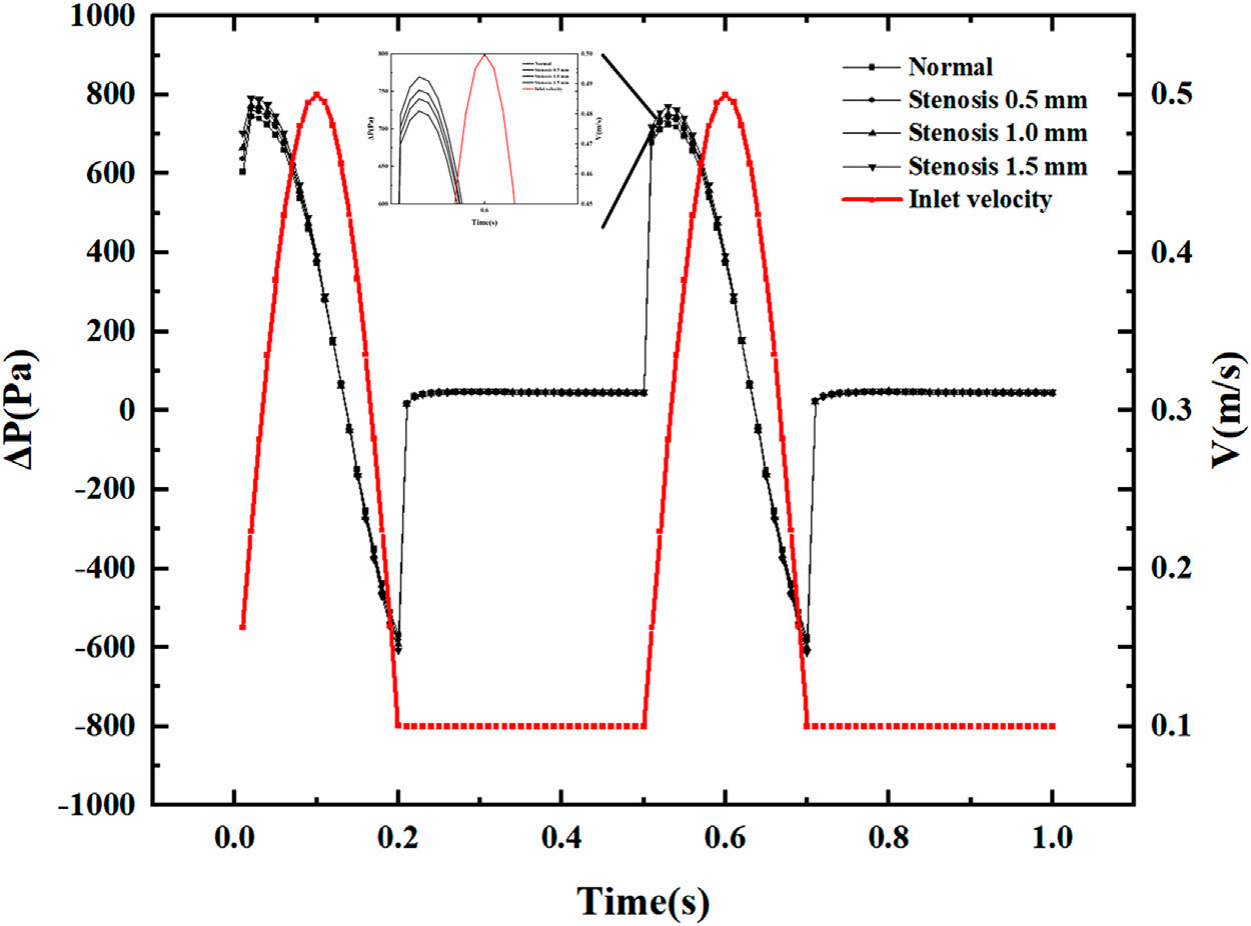
Pressure drop with a pulsation period.

Figure 4 displays pressure drop varies with the pulsation cycle, and the pressure drop fluctuates between −600 and 800 Pa. This pressure fluctuation occurs only during the period when the speed generates pulsation. Within a single pulsation cycle, the pressure drop fluctuation does not show a monotonically increasing or decreasing trend. Still, it produces a slight increase in pressure drop when the inlet velocity increases, and the pressure drop decreases rapidly after reaching the peak pressure drop. The pressure drop is always positive during the inlet velocity increase phase. The pressure drop shows negative values when the velocity reaches its peak and decays. Under constant inlet velocity conditions, the pressure drop is small and has little effect on the pressure drop after stenosis. After the vessel becomes narrowed due to thrombus blockage, the most significant change in pressure drop is in the peak pressure drop region. Each 0.5 mm of narrowing causes an increase in pressure drop of about 20 Pa in the peak pressure drop region. Within a pulsatile cycle, there are two significant sudden pressure drop change points, one where the inlet velocity increases from a constant velocity and the other where the inlet velocity decreases and remains constant.

### 3.2 Wall shear stress distribution.

**FIGURE 5:**
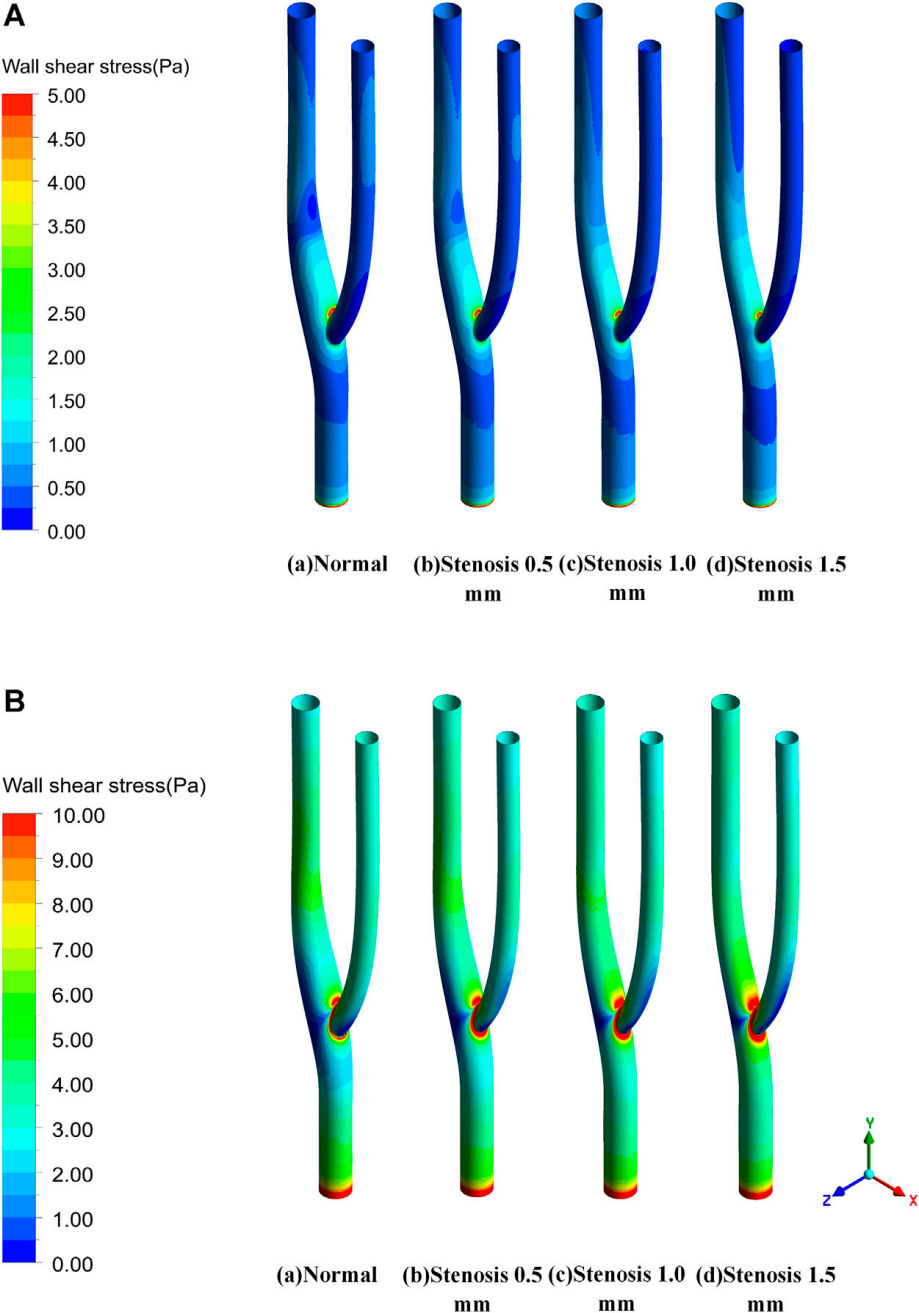
Wall shear stress distribution. **(A) **Wall shear stress distribution at minimum velocity. **(B) **Wall shear stress distribution at maximum velocity.

The blood flow within the artery was simulated using the intra-arterial Carreau model with different degrees of stenosis representing the thickness of the thrombus formed at the arterial bifurcation. Figure 5 shows the wall shear stress distribution for various degrees of stenosis in the bifurcated vessel at maximum and minimum velocity. Under the minimum inlet velocity, the maximum wall shear stress area is concentrated in the inner measurement of the vessel bifurcation. In contrast, the stress will be more minor on the outer side of the arterial bifurcation. After the vessel is narrowed, there is little effect on the wall shear stress upstream of the bifurcation. In contrast, downstream of the bifurcation leads to a gradual concentration of wall shear stress downstream toward the inner measurement of the bifurcation, resulting in a slight decrease in wall shear stress downstream of the bifurcation. Under maximum wall entrance velocity, the wall shear stress is relatively uniform both upstream and downstream of the bifurcation. In contrast, at the arterial bifurcation, the wall shear stress in the inner measurement of the bifurcation is significantly greater than that in other regions. In comparison, the wall shear stress in the outer part of the bifurcation is considerably less than that in other regions. As the vessel becomes progressively narrower, it is evident that the area of low wall shear formed on the lateral side of the bifurcation is progressively larger, which continuously contributes to the formation of a thrombus at the arterial bifurcation.

**FIGURE 6:**
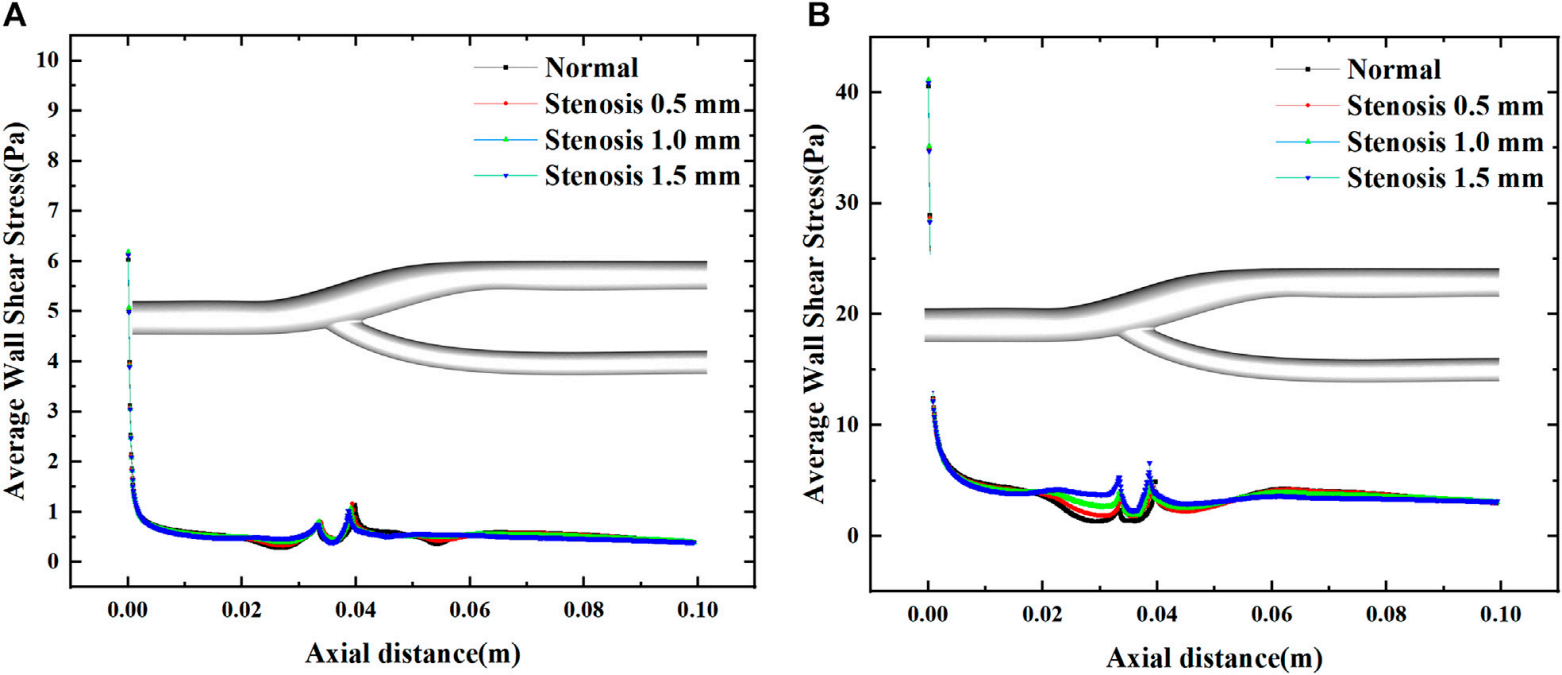
Effect of stenosis on the average wall shear stress along the axial direction. **(A)** Average wall shear stress at minimum velocity. **(B)** Average wall shear stress at maximum velocity.

Figure 6 illustrates the distribution of the mean wall shear stress along the axial direction of the bifurcated segment of the vessel. At maximum and minimum velocities, two abrupt regions of mean wall shear stress are evident at the bifurcation of the artery, mainly due to changes in the geometric surface. For arterial bifurcation, narrowing increases the mean wall shear stress upstream, while downstream of the bifurcation, the effect will be minimal after the bifurcation.

**FIGURE 7:**
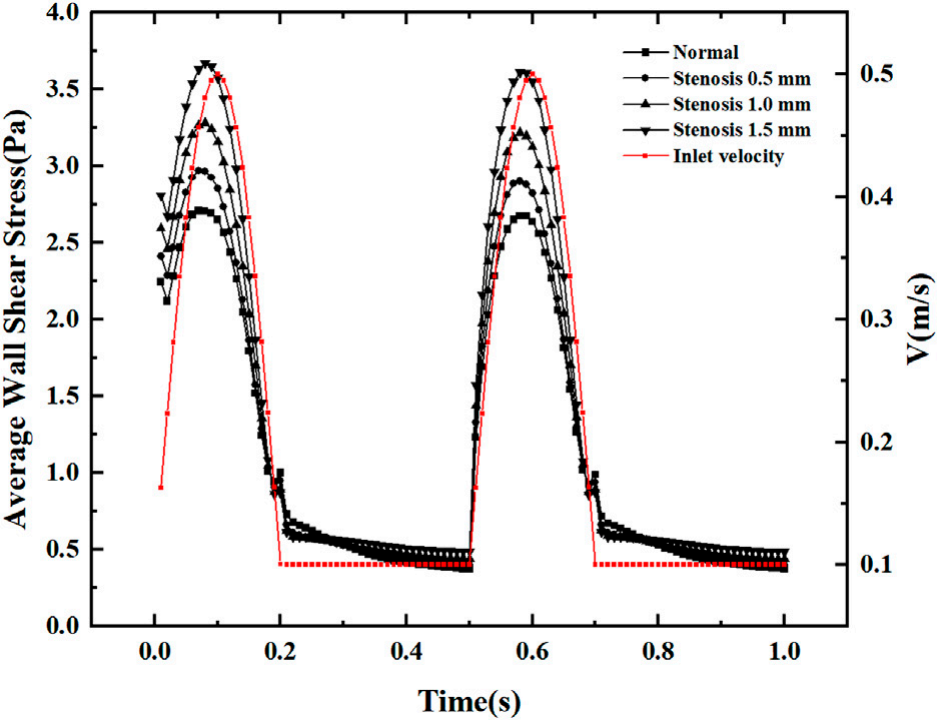
Average wall shear stress variation with a pulsation period.

Figure 7 shows the variation of the mean wall shear stress with the inlet velocity configuration. The variation of the mean wall shear stress is consistent with the variation of the inlet velocity. With the increase of the stenosis, the mean wall shear stress gradually increases during the velocity fluctuation. It tends to be constant during the uniform velocity flow and slightly decreases with time. The mean wall shear stress at the peak velocity increased by about 0.25 Pa for every 0.5 mm bifurcation stenosis, indicating that blood flow pulsation’s effect on the mean wall shear stress mainly existed in the velocity fluctuation region, especially at the peak velocity.

### 3.3 Local heat transfer analysis

**FIGURE 8:**
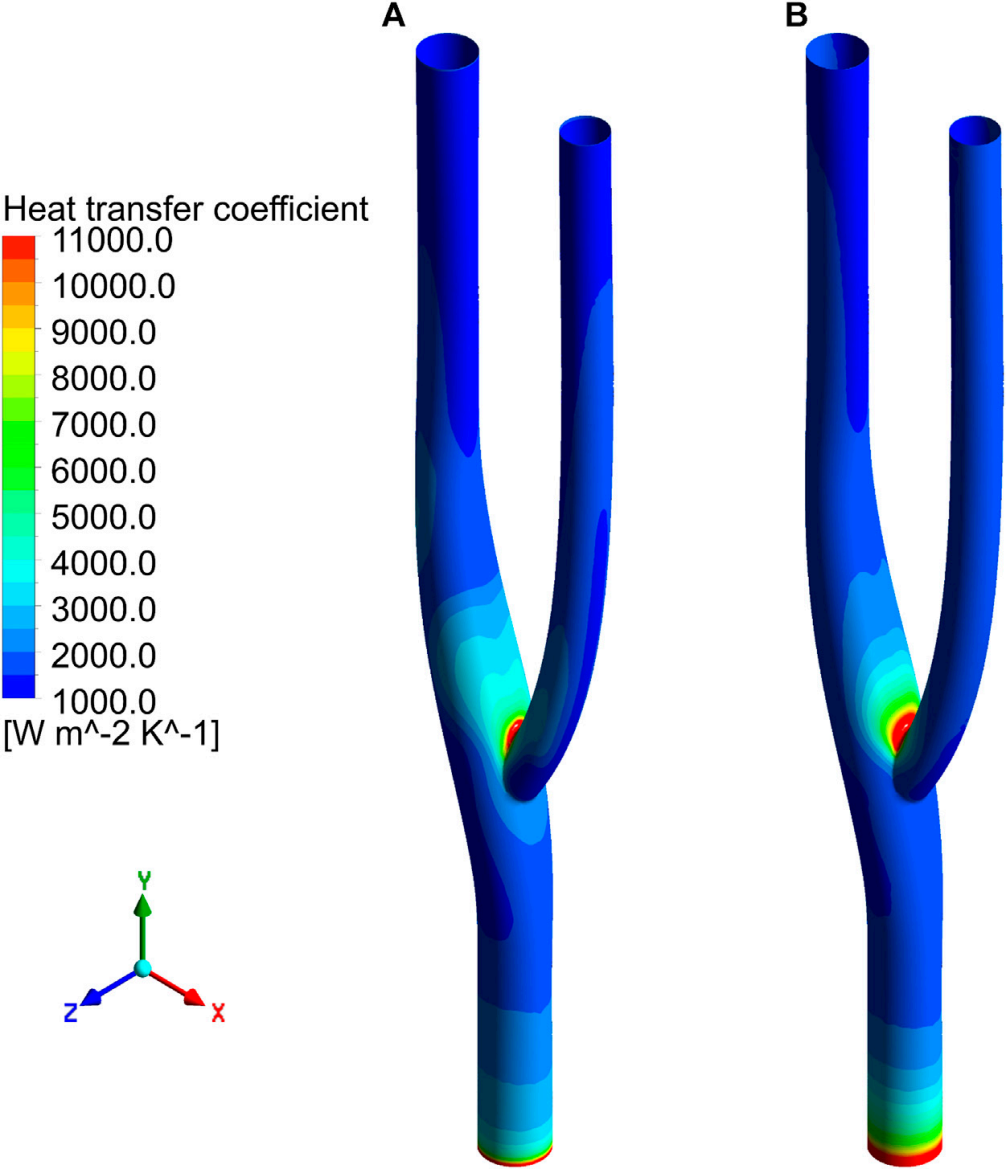
Distribution of surface heat transfer coefficient. **(A)** Heat transfer coefficient distribution at minimum velocity. **(B)** Heat transfer coefficient distribution at maximum velocity.

**FIGURE 9:**
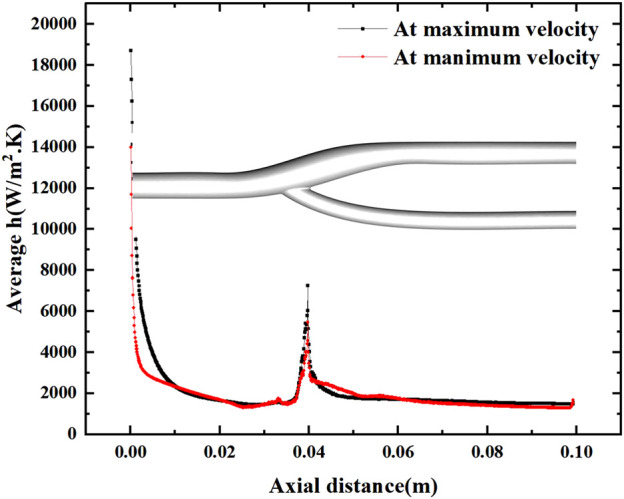
Variation of the average heat transfer coefficient along the axial direction at maximum and minimum velocities.

Figure 8 shows the distribution of the surface heat transfer coefficients of the vascular bifurcation at the minimum and maximum velocities, respectively. In the inner part of the bifurcation, the heat transfer coefficients are significantly more significant than in the other regions. It can also be noted that the main heat transfer region is concentrated in the inner part of the arterial bifurcation, except for the inlet section. In this region, the heat transfer coefficient is several times higher than in the other regions. Figure 9 shows the distribution of the average heat transfer coefficients along the axial direction of the arterial bifurcation at the minimum and maximum inlet velocities. It can be observed that the effect on the heat transfer coefficients at both high and low flow velocities is small, mainly due to the small convective effect of the blood in the vessel, which is primarily unaffected by velocity fluctuations.

**FIGURE 10:**
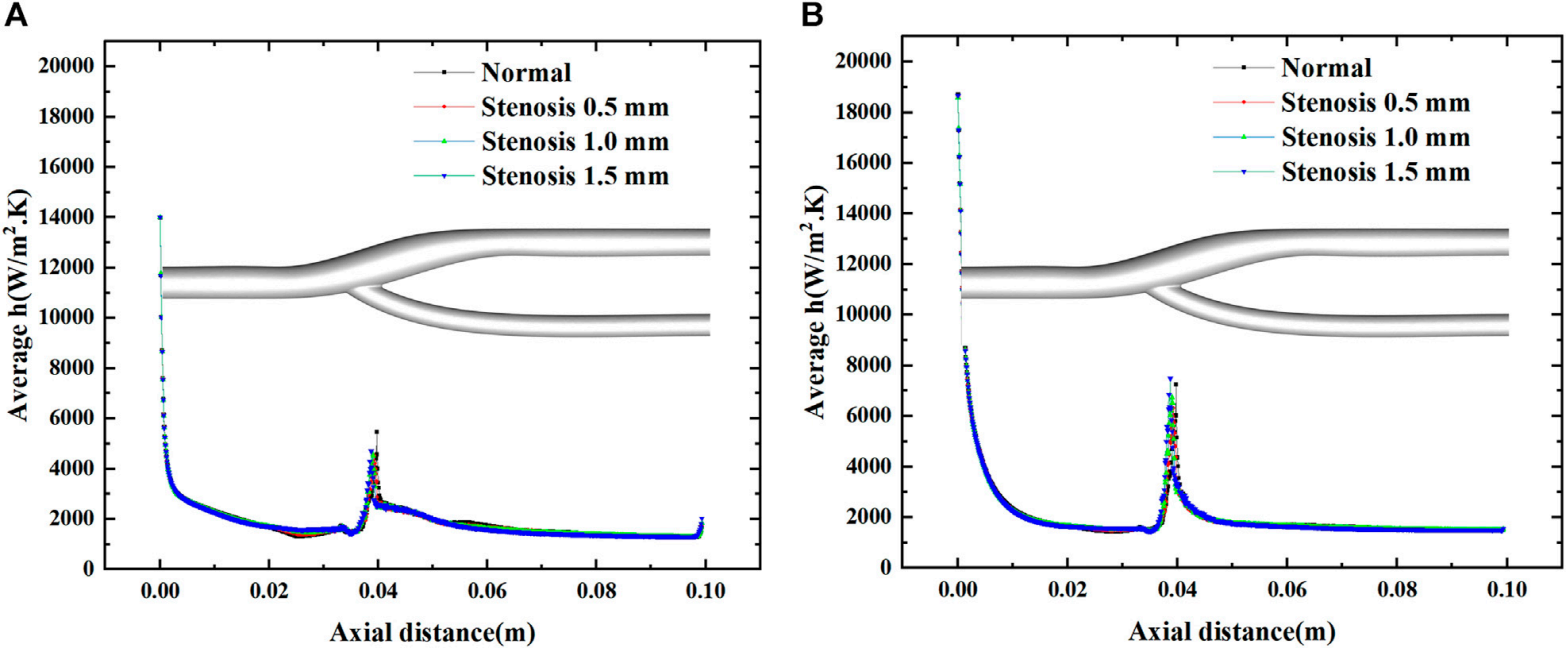
Effect of stenosis on the average heat transfer coefficient along the axial direction. **(A)** Average h at minimum velocity. **(B)** Average h at maximum velocity.

Figure 10 shows the average heat transfer coefficients along the axial direction for different degrees of stenosis at a minimum and maximum velocities. The effect of the degree of stenosis of the vessel bifurcation on the heat transfer of the vessel is fragile and negligible. The average heat transfer coefficient decreases gradually along the axial direction, but the decrease is prolonged. However, there is a pronounced fluctuation at the bifurcation, mainly due to the extensive local flow velocity at the bifurcation, which leads to the local convection enhancement. The convection enhancement in this region is highly correlated with the inlet velocity, and the heat transfer coefficient reaches 4000 W/m2.K at low flow velocity and 6000 W/m2.K at high flow velocity.

**FIGURE 11:**
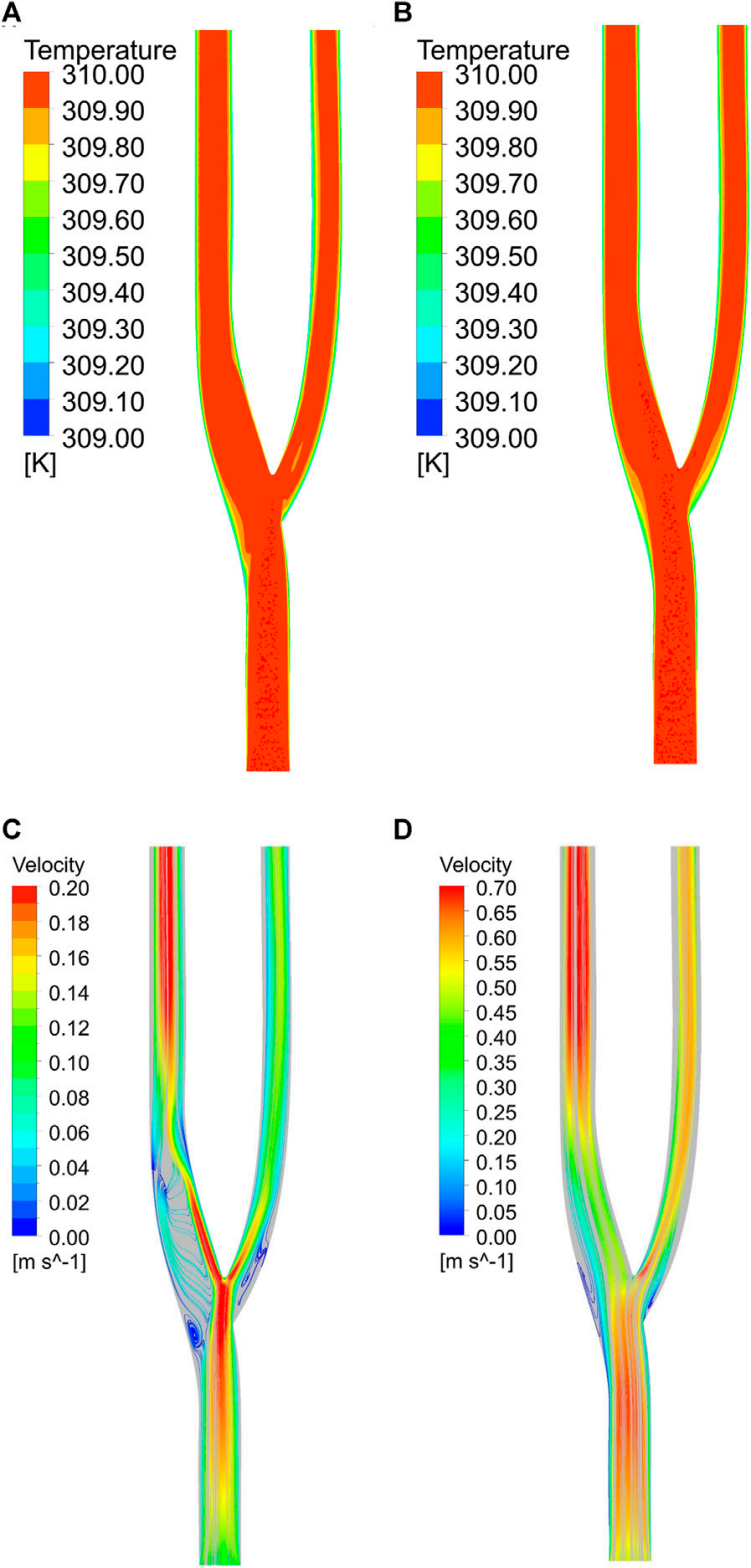
**(A) **Temperature field at minimum velocity. **(B) **Temperature field at maximum velocity. **(C) **Velocity field at minimum velocity. **(D)** Velocity field at maximum velocity.

Figure 11 show the temperature field distribution and velocity field distribution at the minimum and maximum velocities of the normal vessels, respectively. At the minimum velocity, it can be observed that the thermal boundary layer at the bifurcation and downstream of the bifurcation will be thicker, which is detrimental to heat transfer (Lui and Xia, 1998). In contrast, in the velocity field, the velocity field at low flow velocity is not uniform. Two distinct low velocity zones are formed at the outer layer of the bifurcation, in which a reflux effect will be formed. This reflux effect is detrimental to blood flow and will lead to the aggregation of cells in this region, resulting in thrombus formation. A thicker thermal boundary layer exists outside the arterial bifurcation at high flow velocities. Still, the velocity field shows the disappearance of the reflux zone at high flow velocities. In the arterial bifurcation region, the flow velocity is significantly less than the velocity upstream and downstream of the bifurcation.

### 3.4 Flow field analysis

**FIGURE 12:**
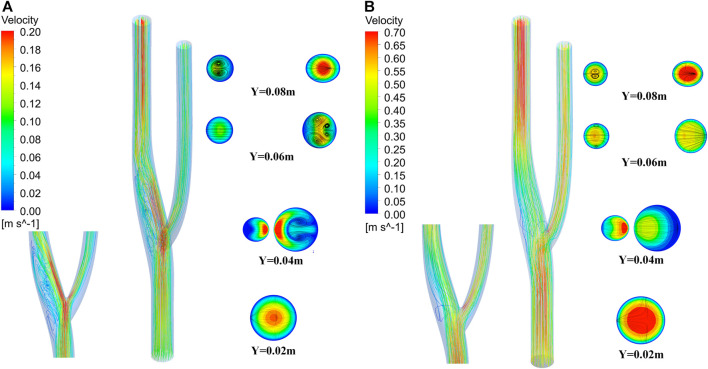
Three-dimensional flow lines and cross-sectional two-dimensional flow lines of vascular bifurcation. **(A) **Streamline at minimum velocity. **(B)** Streamline at maximum velocity.

Figure 12 shows the three-dimensional and two-dimensional streamlines of the cross-section for the vascular bifurcation at the maximum and minimum velocities. The velocity distribution of the cross-section is more uniform at high flow velocity compared to low flow velocity. At low flow velocities, a high velocity flow region and a low velocity flow region are formed at the bifurcation. The high speed region is generated mainly due to the diverging effect of the bifurcation, which leads to the local high speed. And the low velocity region is formed due to the local vortex and the backflow effect of the bend. An apparent high-speed flow region is formed at the main vessel after the bifurcation, and the flow velocity distribution at the bifurcated main vessel is its unevenness, which leads to the vortex flow along the axial direction of the main vessel. At Y = 0.04 m, the velocity distribution at the main vessel was not uniform, and a pair of vortices was formed here, which developed into two teams of Dean vortices at Y = 0.06 m (Kalpakli et al., 2012), while at Y = 0.08 m, the vortices at the main vessel dissipated and a pair of vortices was formed at the secondary vessel. At high velocity flow, vortices never appeared in the main vessel, while vortices started to develop at Y = 0.06 m in the secondary vessel. Still, vortices formed from the boundary, mainly due to the change of flow direction after flowing through the bend.

**FIGURE 13:**
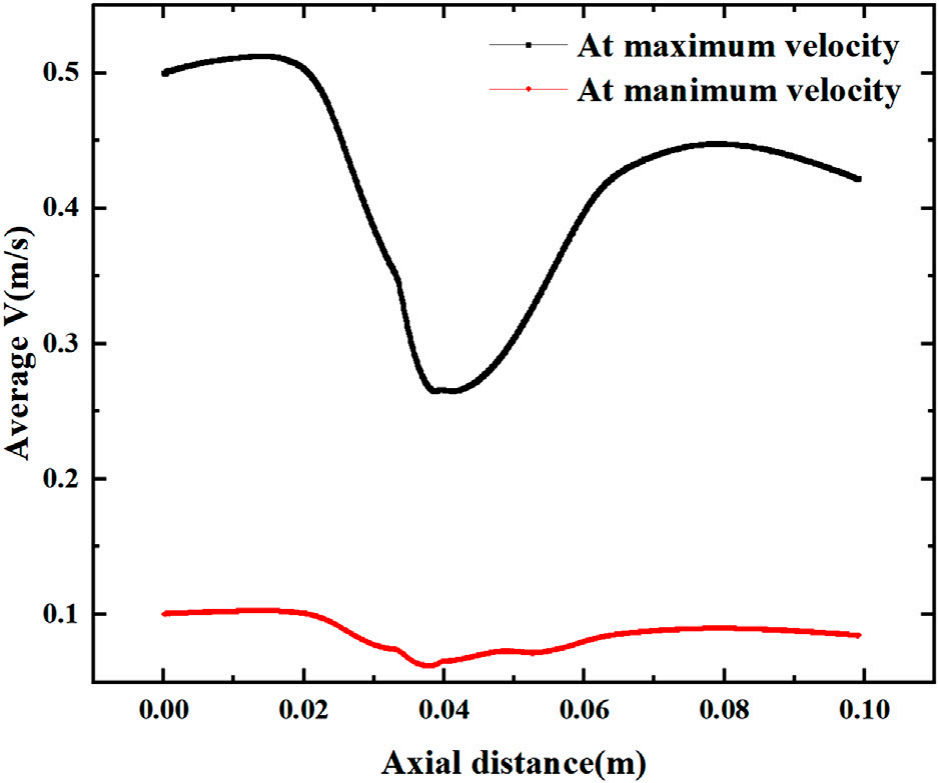
Average velocity variation along the axial direction at maximum and minimum velocities.

Figure 13 shows the mean velocity along the axial direction, where a low velocity segment is formed at the vessel bifurcation (Y = 0.02–0.06 m), which produces a sudden decrease in the blood flow velocity before the bifurcation and another increase in velocity as it flows through the bifurcation. This sudden change in velocity is directly related to the inlet velocity configuration, where the average velocity of flow through the bifurcation decreases by more than 0.2 m/s under the maximum velocity and reduces by about 0.04 m/s under the low inlet velocity profile.

**FIGURE 14:**
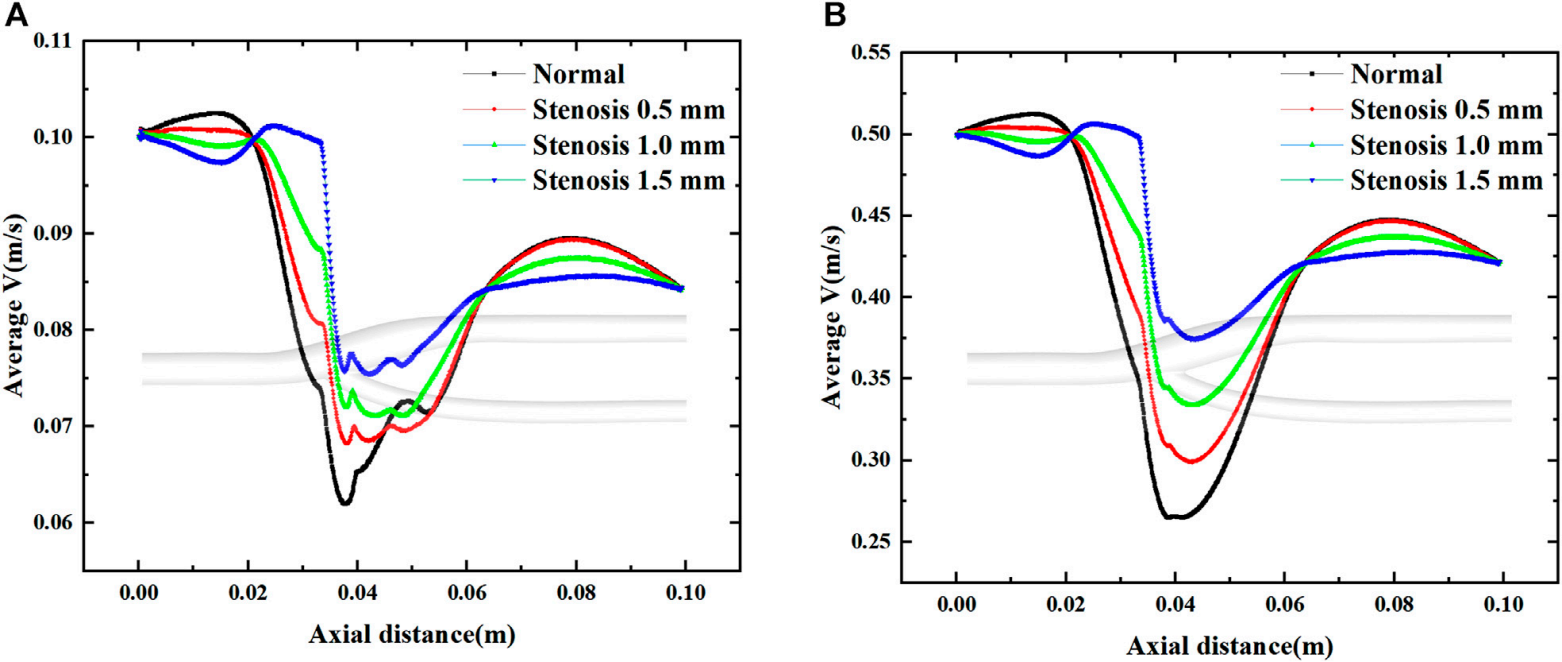
Effect of stenosis on the average velocity along the axial direction. **(A) **Average velocity at minimum velocity. **(B) **Average velocity at maximum velocity.

Figure 14 shows the effect of stenosis on the mean velocity distribution along the axial direction. The effect of stenosis on the bifurcation of the vessel is reflected upstream of the bifurcation (0–0.02 m), at the bifurcation (0.02–0.065 m) and downstream of the bifurcation (0.065–0.1 m). At the upstream and downstream, the narrowing at the bifurcation leads to a decrease in the average axial velocity. In contrast, local stenosis at the bifurcation leads to reduced fluctuations in the mean velocity of the bifurcated segment, but increases the gradient of velocity variation along the axial direction. This is detrimental to blood transport through the bifurcation, and the increase in axial velocity variation increases the pressure on the vessel during pulsation. The decrease in velocity fluctuations, which results in less wall shear stress generated by blood at the bifurcation, is also detrimental to blood flow transport. Especially at high flow velocities, the change in axial velocity is more pronounced in vascular stenosis, with the average axial velocity at the bifurcation increasing by 0.05 m for every 0.5 mm of vessel stenosis, and it is clear that such a change is not negligible.

### 3.5 Longitudinal vortex analysis

**FIGURE 15:**
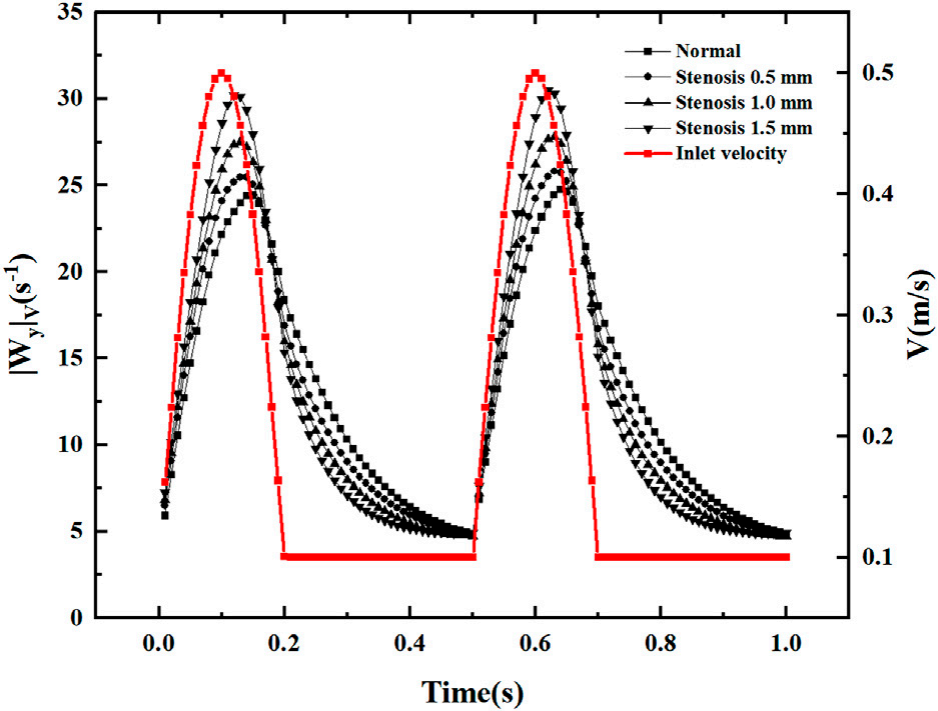
Volume-averaged absolute vorticity with a pulsation period.

After flowing through the bifurcation section, local vortices and secondary flows are inevitably generated. In the current study, volume-averaged absolute vorticity is used to quantify the vortices in the fluid domain (Lin et al., 2009). As shown in Figure 15, the volume-averaged vorticity is accompanied by a periodic variation of the inlet velocity. In the region of increasing velocity, the volume-averaged absolute vorticity increases. When the velocity reaches its peak, the velocity shows a decreasing trend, and the maximum value of volume-averaged absolute vorticity appears after the velocity peak. The volume-averaged absolute vorticity fluctuates within 5-30s^−1^ throughout the pulsation cycle, producing abrupt changes in volume-averaged vorticity at each node of velocity increase. In the region of inlet velocity variation, an increase in stenosis causes volume-averaged absolute vorticity. In the region of constant velocity, an increase in stenosis causes a decrease in volume-averaged absolute vorticity. The volume-averaged vorticity increases rapidly with increasing inlet velocity, reaches a peak, decays rapidly in the velocity dip region, and decreases slowly at constant velocity.

## 4 Conclusion

In this paper, the non-Newtonian pulsatile flow characteristics of carotid blood are investigated numerically, and CFD numerical simulations of non-constant flow are performed using pulsatile boundary conditions. Combining the flow field analysis and heat transfer characteristics, we reveal the effects of stenosis on pressure drop, wall shear stress, heat transfer coefficient and average velocity of the cross-section during pulsation at the carotid bifurcation and other hemodynamic factors.

(1) The pressure drop at the bifurcation section of the carotid artery fluctuates with the inlet velocity configuration, with the pressure drop changing abruptly to about 800 Pa at increasing velocity, decreasing with decreasing velocity, and finally presenting a negative pressure of about 600 Pa. A 0.5-mm increase in arterial bifurcation stenosis increases the peak pressure drop by about 20 Pa.
(2) Wall shear stress increased with the degree of carotid stenosis, and the wall shear stress in the bifurcation segment was mainly concentrated in the inner bifurcation measurement of the artery, while the wall shear stress in the outer part of the bifurcation was smaller. The wall shear stress is more uniformly distributed when the inlet velocity is higher.
(3) The distribution of the wall heat transfer coefficient is consistent with the distribution of wall shear stress, and the uneven distribution of the heat transfer coefficient can be mainly attributed to the uneven distribution of the thermal boundary layer at the wall due to the secondary flow and reflux effect in the flow process.
(4) Increased stenosis of the arterial bifurcation leads to an increase in the average velocity of the local cross-section, and volume-averaged absolute vorticity is introduced to quantify the longitudinal vortex in the fluid domain; arterial stenosis enhances the longitudinal vortex effect when the velocity is pulsating and weakens the longitudinal vortex effect when the inlet is uniform.

## Data Availability

The original contributions presented in the study are included in the article/supplementary material, further inquiries can be directed to the corresponding authors.
